# *In vivo* imaging of leucine aminopeptidase activity in drug-induced liver injury and liver cancer *via* a near-infrared fluorescent probe†
†Electronic supplementary information (ESI) available. See DOI: 10.1039/c6sc05712h



**DOI:** 10.1039/c6sc05712h

**Published:** 2017-03-02

**Authors:** Xinyuan He, Lihong Li, Yu Fang, Wen Shi, Xiaohua Li, Huimin Ma

**Affiliations:** a Beijing National Laboratory for Molecular Sciences , Key Laboratory of Analytical Chemistry for Living Biosystems , Institute of Chemistry , Chinese Academy of Sciences , Beijing 100190 , China . Email: shiwen@iccas.ac.cn ; Email: mahm@iccas.ac.cn; b University of the Chinese Academy of Sciences , Beijing 100049 , China

## Abstract

The liver, a main detoxification organ, has evolved a complex enzymatic system to respond to multiple pathological conditions, in which leucine aminopeptidase (LAP) has been reported to participate in detoxifying cisplatin in hepatoma cells and contribute to the intrinsic drug resistance. *In vivo* imaging of LAP activity in liver disease models is thus helpful to further understand the function of LAP in detoxification and medicine, but such an imaging approach is still lacking. Herein, we develop a selective and sensitive near-infrared fluorescent probe (HCAL) for this purpose. Using the probe, combined with confocal fluorescence imaging, we disclose the upregulations of LAP in acetaminophen-induced liver injury and tumor-bearing mice models. Supplementary acetylcysteine can suppress this upregulation, revealing that the LAP increase may be connected with a deficiency in biothiols. Moreover, HCAL has been used to image LAP in hepatoma cells, tumor tissues and xenograft tumor mice models successfully. These results demonstrate that HCAL may be a promising tool for studying the function of LAP in LAP-associated liver diseases.

## Abstract 2

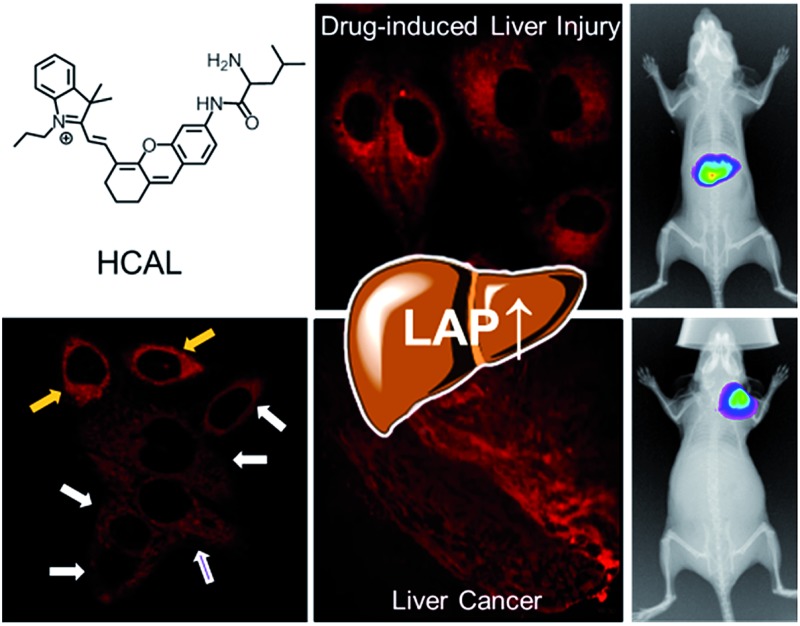
The upregulation of leucine aminopeptidase in hepatopathy models is imaged *in vivo* for the first time with a near-infrared fluorescent probe.

## Introduction

The human body is exposed to a wide array of toxic substances in one’s lifetime, including environmental toxins and pharmaceuticals, and thus has evolved a complex detoxification system.^1^ Current evidence suggests an association between an impaired detoxification system and various diseases, such as cancer, fibromyalgia, and chronic fatigue/immune dysfunction syndrome.^2^ The majority of detoxification occurs in the liver, one of the most toxin-sensitive organs.^3^ For example, nontoxic acetaminophen (Ace), a routine analgesic and antipyretic drug, can be converted to the toxic *N*-acetyl-*p*-benzoquinone imine in the liver by cytochrome P-450 enzymes.^4^ This metabolite is then detoxified through conjugation with glutathione (GSH). An overdose of Ace generates a large amount of *N*-acetyl-*p*-benzoquinone imine, which may deplete the amount of intracellular thiols, thereby leading to liver cell damage. To minimize the potential damage, the liver has developed a complex enzymatic system to respond to pathological conditions.^5^ Accordingly, variations in the amount of related enzymes can be used as indicators of liver dysfunction, among which leucine aminopeptidase (LAP; EC 3.4.11.1) has recently been reported to contribute to the intrinsic resistance of cancer cells towards cisplatin by our group.^6^ Hepatoma cells with a higher level of LAP have stronger resistance toward cisplatin than those with a lower level of LAP.^6^ This implies that LAP may participate in detoxifying cisplatin in hepatoma cells. Obviously, *in vivo* imaging of LAP activity in liver disease models is helpful to further understand the physiological function of LAP in detoxification, but such an imaging approach is still lacking. Our previous LAP probe showed good performance in imaging LAP in cells, but its emission wavelength is not long enough to meet the demand for *in vivo* imaging. In this respect, fluorescent probes with near-infrared (NIR) emission (>650 nm) have significant advantages such as deep tissue penetration and minimum background autofluorescence.^7^ So far, however, no NIR probe has been used for imaging LAP *in vivo*, which propelled us to develop a superior NIR fluorescent probe for *in vivo* imaging of the LAP variation in liver disease models.

**Fig. 1:**
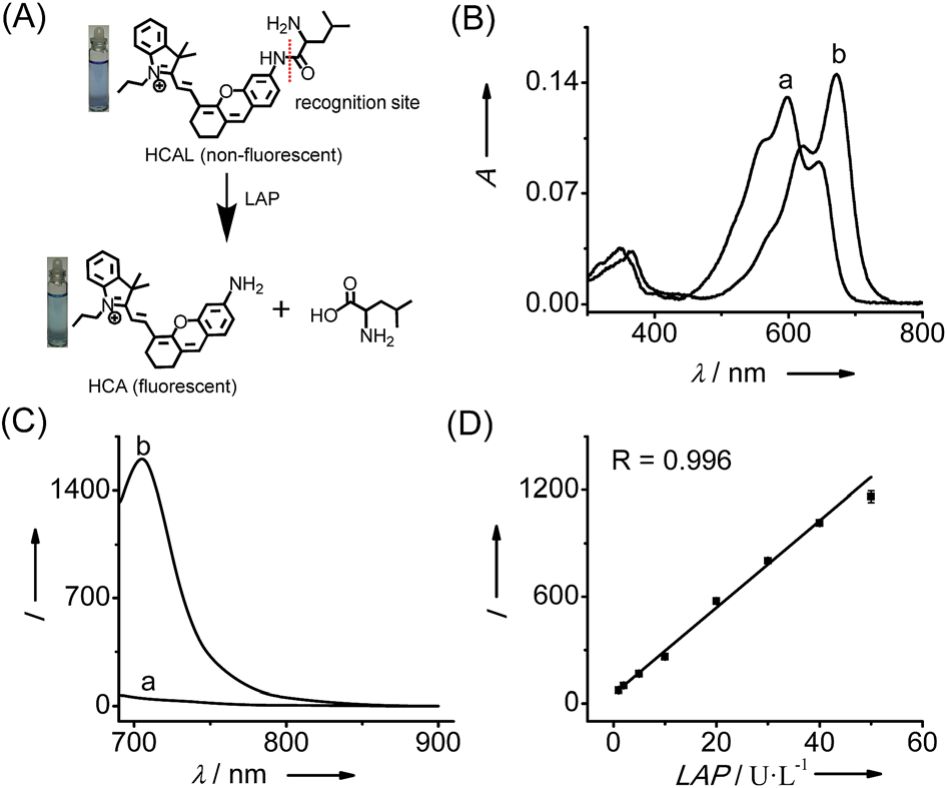
(A) Structure of HCAL and its reaction with LAP. (B) Absorption and (C) fluorescence emission spectra of HCAL (5 μM) before (a) and after (b) reaction with LAP (100 U L^–1^) at 37 °C in pH 7.4 PBS for 90 min. (D) Linear fitting curve of the fluorescence intensity towards the concentration of LAP from 1–50 U L^–1^. *λ*_ex/em_ = 670/705 nm.

Based on the ability of LAP to hydrolyze N-terminal leucyl groups, we designed such a probe, HCAL (Fig. 1A and Scheme S1†), by conjugating the amino group of a NIR hemicyanine with the carboxyl group of leucine. Stable hemicyanines with a hydroxyl group have recently been continually employed for preparing NIR probes,^8^ but those with an amino group are seldom available except for some recent examples.^9^ Here, we have successfully synthesized an amino hemicyanine (HCA) *via* two steps (Scheme S1†), and found that, similar to a hydroxyl hemicyanine,^8^ amino substitution by leucine can efficiently quench the hemicyanine’s fluorescence, producing an extremely low background fluorescence, which is expected to afford high sensitivity.

## Results and discussion

### Spectroscopic response of probe HCAL to LAP

Fig. 1 shows the spectroscopic properties of the probe HCAL and its response to LAP. HCAL itself displays a strong absorption peak at 598 nm and rather weak fluorescence (quantum yield *Φ* < 0.01 in PBS). Upon the addition of LAP, however, the maximum absorption peak is red-shifted to about 670 nm, accompanied by a distinct color change from light blue to cyan (Fig. 1A) and a large fluorescence enhancement (32-fold) at 705 nm. Moreover, the absorption and fluorescence spectra of the reaction system coincide well with those of the fluorophore HCA (Fig. S7†), implying the release of HCA (*Φ* = 0.11 in PBS) from the reaction system. Electrospray ionization mass spectral and HPLC analyses of the reaction products further verified the hydrolysis of the probe and the generation of HCA. As depicted in Fig. S8 (ESI†), a mass peak at *m*/*z* = 411.4 [M]^+^, characteristic of HCA, is detected in the reaction solution, and a new chromatographic peak at 10.56 min is generated (Fig. S9†), which is in accordance with that of HCA.

The analytical conditions, including pH, temperature and reaction time for the LAP assay, were studied. As shown in Fig. S10 (ESI†), the fluorescence of HCAL itself is not largely affected by the change in pH from 5.6 to 9.1 in PBS and temperature from 25 to 42 °C, and upon reaction with LAP a maximum fluorescence enhancement can be achieved at about pH 7.4 and 37 °C, suggesting the excellent performance of the probe under normal physiological conditions. Moreover, the fluorescence enhancement initiates immediately and reaches a plateau in about 90 min. Thus, all of the following measurements were conducted in PBS of pH 7.4 at 37 °C for 90 min. In addition, both HCAL and HCA displayed good photostability under the irradiation of a xenon lamp (Fig. S10D†). Under the optimized conditions, the probe HCAL was titrated with LAP, and the fluorescence enhancement of the reaction system exhibited good linearity with an equation of *I* = 24.4 × *C* (U L^–1^) – 0.6 (*R* = 0.996) in the concentration range of 1–50 U L^–1^ for LAP (Fig. 1C). The detection limit (*k* = 3)^10^ was found to be 0.19 U L^–1^, and the Michaelis constant of the probe towards LAP was determined to be 123 μM (Fig. S11†), indicating a strong affinity towards LAP for the probe.

The selectivity of HCAL for LAP was examined over other potential coexisting species, such as inorganic salts (KCl, MgCl_2_, CaCl_2_ and CuSO_4_), biomolecules (vitamin C, vitamin B6, glucose, cysteine, glutathione, glutamic acid, alanine, arginine and glycine), reactive oxygen species (NaOCl and H_2_O_2_) and some similar aminopeptidases. As shown in Fig. S12 (ESI†), the probe displayed high selectivity towards LAP, which is attributed to the specific cleavage of the leucyl group by LAP. To further confirm the LAP-catalyzed cleavage, bestatin, a specific inhibitor of LAP,^11^ was added to the reaction system. As depicted in Fig. S13 (ESI†), 1 μM of bestatin decreases the fluorescence by 59%, and more bestatin (10 μM) produces a larger decrease (80%) compared to the control group without bestatin. Furthermore, the fluorescence of both the probe and its product HCA does not change significantly with the addition of bestatin (Fig. S13B†). Thus, the fluorescence off-on response of the probe toward LAP is indeed caused by enzymatic hydrolysis.

### Detection of LAP in living cells

**Fig. 2:**
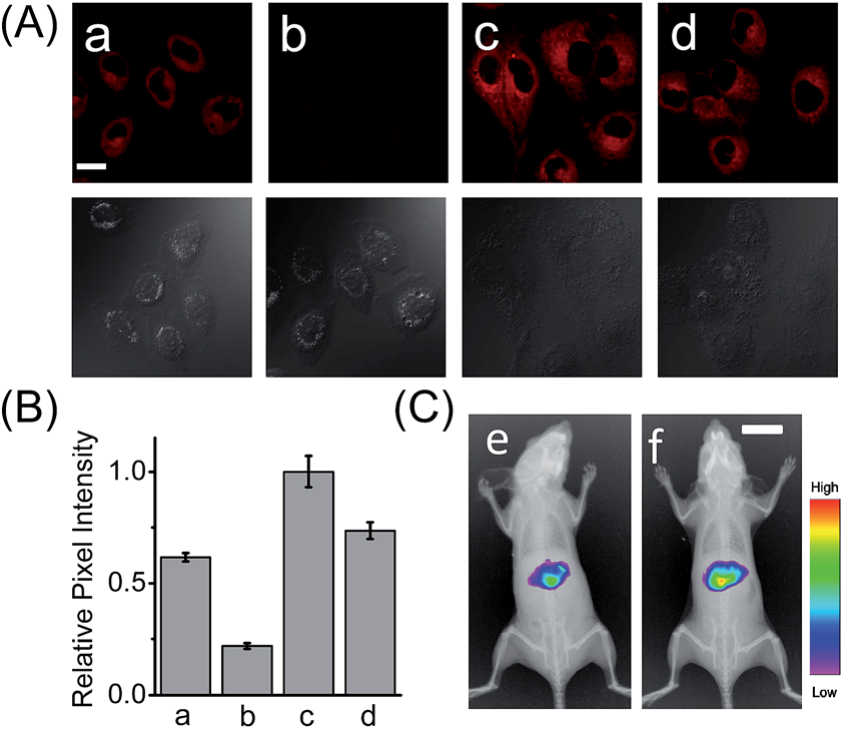
(A) Confocal fluorescence images of the LO2 cells with HCAL. (a) Intact LO2 cells; (b) LO2 cells pretreated with bestatin (100 μM) for 1 h; (c) LO2 cells pretreated with Ace (2 mM) for 48 h; (d) LO2 cells pretreated with Ace (2 mM) in the presence of acetylcysteine (100 μM) for 48 h. Scale bar: 20 μm. The differential interference contrast (DIC) images of the corresponding sample are shown below. (B) The relative pixel intensity of the corresponding fluorescence images in panel A. (C) *In vivo* fluorescence imaging of mice that were intraperitoneally preinjected with (e) PBS and (f) 300 mg kg^–1^ Ace before intravenous injection of 100 μL HCAL (200 μM) in PBS. Scale bar: 1 cm.

HCAL exhibits good biocompatibility, since the probe even at 15 μM did not produce a considerable influence on the viability of the cells (Fig. S14†). Next, we examined the capability of the probe to image the endogenous LAP in a human normal hepatocyte cell line (LO2). As shown in Fig. S15 (ESI†), when LO2 cells were incubated with HCAL, the fluorescence in the cells gradually increased and reached a plateau in about 30 min, indicating good cell permeability of the probe and its reaction with the intracellular LAP. Moreover, as depicted in Fig. 2A, under the same imaging condition the bestatin pretreated LO2 cells exhibit a largely suppressed fluorescence as compared to the control without the inhibitor treatment (compare images a and b; see also Fig. 2B). This fluorescence change can be attributed to the alteration of the intracellular LAP activity; in other words, the probe is capable of imaging the endogenous LAP. Interestingly, we noted that the reaction between HCAL and LAP in the cells is much faster than that *in vitro* (compare Fig. S10C and S15†), which might be ascribed to the higher activity of LAP in its natural form in the cells.

### Detection of LAP in drug-induced liver injury model

Having demonstrated its excellent analytical properties, the probe was then used to study the changing behavior of LAP in liver cells under different pathological conditions, using confocal fluorescence microscopy. As mentioned above, the liver, a central organ in the metabolism and detoxification of drugs, is more vulnerable to toxic damage,^12^ and LAP may play a role in detoxifying a drug in hepatoma cells.^6^ To gain an insight into the role of LAP, using HCAL, we monitored the alteration of LAP in a drug-induced liver injury model.^13^ Ace, as a drug, was employed to simulate such an injury, since its concentration up to 5 mM has no effect on the fluorescence of HCAL, HCA and LO2 cells or on the morphology of the cells (Fig. S16†). As depicted in Fig. S17 (ESI†), the Ace treated LO2 cells show a dose- and time-dependent enhancement in fluorescence and this increase can be significantly inhibited by bestatin, which reveals an upregulation of LAP in the drug-induced liver injury model. This upregulation was further verified by western blot analysis (Fig. S18†). The possible reason for this phenomenon might be that excessive amounts of the metabolites of Ace, such as *N*-acetyl-*p*-benzoquinone imine, over-consume the intracellular GSH, and thus cause a significant increase in the oxidative stress, which is harmful to the cells. On the other hand, to antagonize the increase in the oxidative stress, the cells themselves have to recruit more GSH. Interestingly, it is known that LAP has cysteinyl-glycine hydrolysing activity in the liver and can accelerate the production of supplementary cysteine (a rate-limiting factor in GSH synthesis).^14^ Hence, we may propose that the upregulation of intracellular LAP might be an instinctual response to GSH consumption and cell injury. This mechanism may partially explain our previous observation that cells with a higher LAP level have a higher intrinsic resistance to cisplatin.^6^ To further confirm the mechanism, a comparative study was made by imaging LO2 cells that had been pretreated with Ace in the absence and presence of acetylcysteine, which is an efficient cysteine supplement for GSH synthesis.^15^ As shown in Fig. 2A, the fluorescence from the Ace treated cells in the presence of acetylcysteine (image d) is significantly depressed as compared to the control in the absence of acetylcysteine (image c; see also Fig. 2B), verifying that the upregulation of LAP in the drug-induced liver injury model is closely associated with the level of biothiols.

Furthermore, we used HCAL to image the LAP activity in Ace overdosed mice *in vivo*.^16^ As depicted in Fig. 2C, after the mice were successively subjected to an intraperitoneal injection of pH 7.4 PBS (control) and an intravenous injection of the probe, the liver region displayed remarkable fluorescence (image e), which accords with the fact that the liver contains plenty of LAP;^17^ however, if the mice were intraperitoneally injected with 300 mg kg^–1^ Ace before the probe injection, the mice exhibited stronger fluorescence (image f) than the control, also implying the upregulation of LAP in the drug-induced liver injury model. This is the first probe that can image the LAP activity *in vivo* to the best of our knowledge.

### Detection of LAP in a liver cancer model

**Fig. 3:**
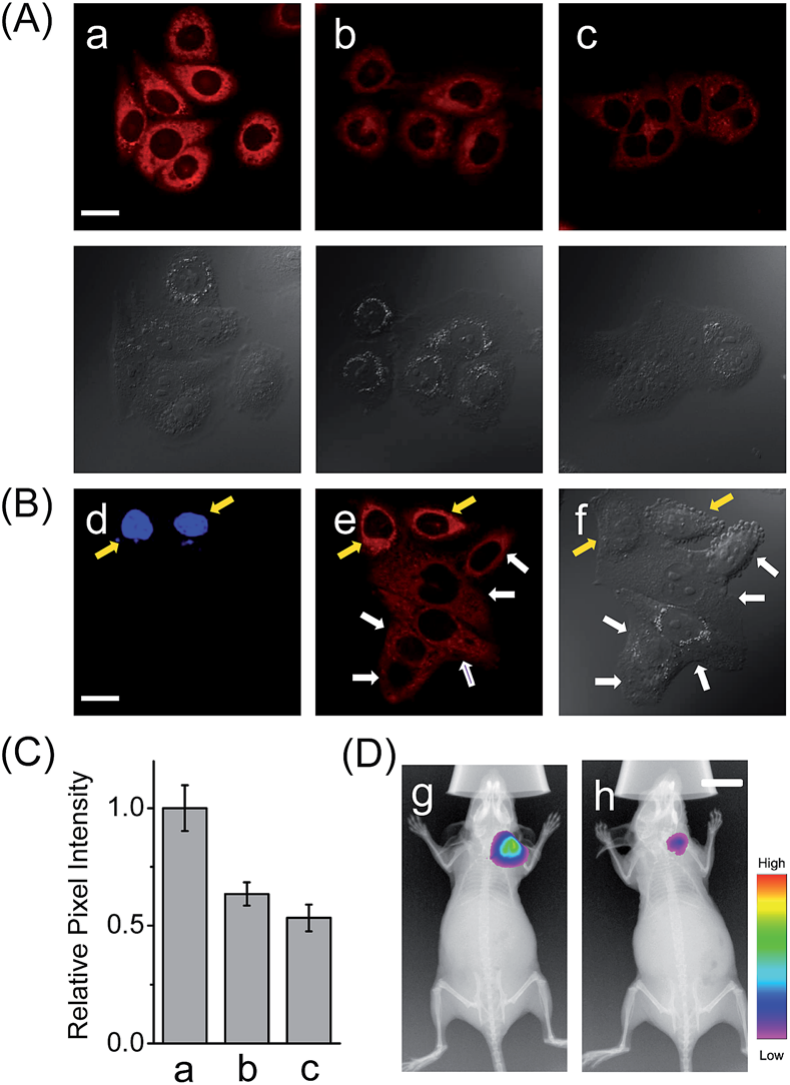
(A) Confocal fluorescence images of cells with the probe HCAL. (a) HepG2 cells; (b) LO2 cells; (c) HepG2 cells pretreated with bestatin (100 μM) for 1 h. The DIC images of the corresponding sample are shown below. Scale bar: 20 μm. (B) Confocal fluorescence images of the cell mixture (LO2 and Hoechst-33342 stained HepG2 cells) with the probe HCAL. Image d: *λ*_ex_ = 405 nm; image e: *λ*_ex_ = 635 nm; image f: the corresponding DIC image. The yellow arrows indicate the HepG2 cells which were pre-stained with Hoechst-33342, and the white arrows indicate the LO2 cells. Scale bar: 20 μm. (C) The relative pixel intensity of the corresponding fluorescence images in panel A. (D) *In vivo* fluorescence imaging of HepG2 tumor xenografted mice that were intratumorally preinjected with 50 μL of (g) PBS (control) and (h) bestatin (100 μM in PBS) for 1 h before intratumoral injection of 50 μL HCAL (50 μM) in PBS. Scale bar: 1 cm.

In addition to the drug-induced liver injury model, LAP also correlates with the prognosis and malignant development of hepatocellular carcinomas.^18^ Therefore, the probe HCAL was further utilized to image the LAP activity in cancer cells and tissues, as well as in tumor-bearing mice *in vivo*. When hepatocarcinoma cell line HepG2 cells and normal hepatocyte cell line LO2 cells were treated with HCAL under the same conditions, HepG2 cells exhibited a much higher fluorescence intensity than LO2 cells (Fig. 3A), and the strong fluorescence in the HepG2 cells can be suppressed by bestatin (see also Fig. 3C). This suggests that the fluorescence difference between the HepG2 and LO2 cells reflects the relative level of intracellular LAP, as clearly confirmed by western blot analysis (Fig. S19†). Notably, when the HepG2 and LO2 cells were cultured in one dish and then incubated with HCAL, the two cell lines were easy to differentiate through the different fluorescence intensity of HCAL in the red channel (image e in Fig. 3B), which is further supported by the Hoechst-33342 pre-stained method (blue channel; image d in Fig. 3B). Inspired by these results, we moved forward to investigate the ability of HCAL in imaging LAP activity in tissue sections, which is very important for cancer diagnosis. Tumor tissues were harvested from a HepG2 xenograft tumor model. The specimens were cut using a cryostat microtome at a thickness of 5 μm. Three adjacent sections were subjected to HCAL, immunohistochemical and hematoxylin-eosin staining, respectively. As shown in Fig. S20,† the fluorescence image induced by the LAP activity is in good accordance with the immunohistochemical staining image, but exhibits a much clearer boundary, which indicates the distribution of LAP in the different cancer regions, as identified by the hematoxylin-eosin staining. Furthermore, the probe HCAL was employed to image the LAP activity in tumor-bearing mice *in vivo*. As shown in Fig. 3D, after the HepG2 tumor-bearing mice were intratumorally injected with HCAL, a strong fluorescence was observed in the tumor region (image g). Moreover, this strong fluorescence may last at least for 4 h (Fig. S21†), which might be helpful for surgical cancer treatment. Under the same imaging conditions, however, the tumor-bearing mice which were pretreated with bestatin for 1 h displayed a much weaker fluorescence in the tumor region (image h in Fig. 3D and S21†), which validates that the fluorescence of the tumor indeed arises from the LAP activity. Taken together, the above observations demonstrate that HCAL is useful for distinguishing normal liver cells from hepatoma cells based on their distinctive LAP activity.

## Conclusions

In summary, we have developed HCAL as a NIR fluorescent off-on probe for the specific detection of LAP with a detection limit of 0.19 U L^–1^. The probe has been used to image the LAP activity in a drug-induced liver injury model and in tumor-bearing mice *in vivo*. It is found that LAP can be upregulated in both of the two liver disease models, revealing the importance of LAP in liver function and its potential value in hepatopathy diagnosis and therapy. Additionally, due to its NIR features, the probe HCAL may serve as a promising tool for research on LAP-associated diseases.
