# Supplementary material for: In vivo imaging of leucine aminopeptidase activity in drug-induced liver injury and liver cancer via a near-infrared fluorescent probe
Source: Chem Sci. 2017 Mar 2;8(5):3479–83. doi: 10.1039/c6sc05712h (PMC5418645; doi:10.1039/c6sc05712h)
Supplement: Supplementary file 1 [file SC-008-C6SC05712H-s001.pdf]

Electronic Supplementary Material (ESI) for Chemical Science.
This journal is © The Royal Society of Chemistry 2017

Electronic Supplementary Information for

In vivo imaging of leucine aminopeptidase activity in drug-induced liver injury and liver cancer via a near-infrared fluorescent probe

Xinyuan He,[a,b] Lihong Li,[a] Yu Fang,[a] Wen Shi, *,[a,b] Xiaohua Li,[a] and Huimin Ma*,[a,b]

[a] Beijing National Laboratory for Molecular Sciences, Key Laboratory of Analytical Chemistry for Living Biosystems, Institute of Chemistry, Chinese Academy of Sciences, Beijing 100190, China.

[b] University of the Chinese Academy of Sciences, Beijing 100049, China.

Email: shiwen@iccas.ac.cn; mahm@iccas.ac.cn.

Note added after first publication: This Electronic Supplementary Information file replaces that originally published on 2nd March 2016, and contains a corrected Figure S4.

1. Apparatus and reagents

Fluorescence measurements were made on a Hitachi F-4600 spectrophotometer in 10 mm × 10 mm quartz cells (Tokyo, Japan). ^{1}H- and ^{13}C-NMR spectra were measured with a Bruker DMX-400 spectrometer. Electrospray ionization (ESI) mass spectra were measured on a Shimadzu LC-MS 2010A instrument (Kyoto, Japan). High resolution electrospray ionization mass spectra (HR-ESI-MS) were recorded on an APEX IV FTMS instrument (Bruker, Daltonics). Absorption spectra were recorded in 1-cm quartz cells with a TU-1900 spectrophotometer (Beijing, China). MTT [3-(4,5-dimethyl-2-thiazolyl)-2,5-diphenyl-2*H*-tetrazolium bromide] analysis was made on a microplate reader (Molecular Devices SpectraMax i3). Fluorescence imaging was conducted on an FV 1200-IX83 confocal laser scanning microscope (Olympus). Imaging of histological sections was conducted on a BX43 fluorescence microscope (Olympus). In vivo fluorescence imaging was made on a Kodak In-vivo Imaging System FX Pro.

IR-780 iodide, leucine aminopeptidase (LAP), dipeptidyl peptidase-4 (DPPIV), pyroglutamate aminopeptidase (PGP-1), alanine aminopeptidase (APN), bestatin and acetaminophen (Ace) were obtained from Sigma-Aldrich. Boc-L-leucine and $SnCl_2$ were purchased from Beijing InnoChem Science & Technology Co. Ltd. 3-Nitrophenol was purchased from J & K Chemical Ltd. Phosphate buffered saline (PBS: 155 mM NaCl, 2.97 mM Na_2HPO_4, 1.05 mM KH_2PO_4; pH 7.4), Dulbecco's modified Eagle's medium (DMEM), and fetal bovine serum (FBS) were purchased from Thermofisher. All the cell lines used were obtained from KeyGEN BioTECH Co. Ltd. (Nanjing, China). Ultrapure water (over 18 MΩ cm) was employed throughout.

2. Syntheses of compounds

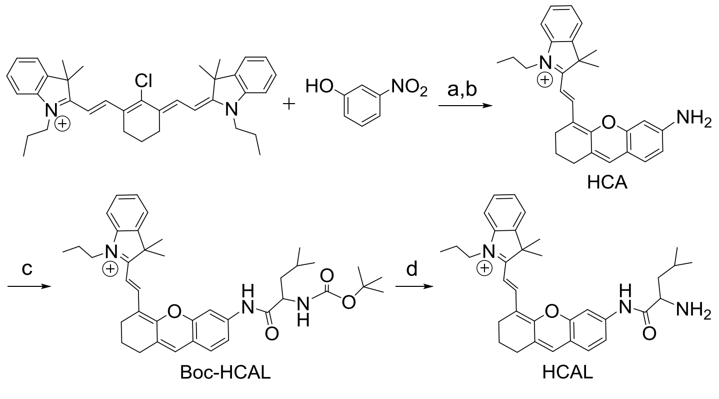

Scheme S1. Synthesis of HCAL. Conditions: a) K_2CO_3, CH_3CN, room temperature, 4 h; b) $SnCl_2$, HCl, CH_3OH, 70 ^{o}C, overnight; c) Boc-L-leucine, HATU, DIPEA, room temperature, 4 h; d) CF_3COOH, CH_2Cl_2, room temperature, 3 h.

Synthesis of HCA. 3-Nitrophenol (347 mg, 2.5 mmol) and K_2CO_3 (345 mg, 2.5 mmol) were dissolved in 15 mL CH_3CN in a flask, and the mixture was stirred at room temperature under argon atmosphere for 10 min. Then, IR-780 iodide (667 mg, 1 mmol) in CH_3CN (2 mL) was introduced to the mixture via a syringe and the reaction mixture was stirred at room temperature for 4 h. The solvent was then evaporated under reduced pressure and the precipitate was dissolved in CH_2Cl_2, followed by washing with water for three times and drying over Na_2SO_4. The residue obtained by evaporation was dispersed in 30 mL CH_3OH for further use in the next step.

$SnCl_2$ (4 g, 20 mmol) was dissolved in concentrated HCl (4 mL), followed by adding the above HCA solution under argon atmosphere. The reaction solution was heated to 70 ℃ and stirred overnight. Then, the solution was neutralized by saturated Na_2CO_3, and the precipitate was removed by filtration and washed with CH_2Cl_2. The collected filtrate and washings were treated thrice with water and dried over Na_2SO_4. The solvent was removed by evaporation under reduced pressure, and the residue was subjected to silica gel chromatography with CH_2Cl_2/CH_3OH (100/1 to 20/1, v/v) as eluent, affording HCA as green solid (260 mg, yield 63%). ^1H and ^{13}C NMR spectra are shown in Figures S1 and S2. ^1H NMR (400 MHz, 298 K, CD_3OD): δ 8.61 (d, 1H, J = 14.4 Hz), 7.56-7.52 (m, 2H), 7.45 (t, 1H, J = 7.2 Hz), 7.38-7.30 (m, 3H), 6.77-6.74 (m, 1H), 6.70 (s, 1H), 6.24 (d, 1H, J = 14.4 Hz), 4.17 (t, 2H, J = 7.2 Hz), 2.77 (t, 2H, J = 6 Hz), 2.70 (t, 2H, J = 6 Hz) 1.96-1.85 (m, 4H), 1.78 (s, 6H), 1.07 (t, 3H, J = 7.2 Hz). ^{13}C NMR (100 MHz, 298 K, CD_3OD): δ 174.4, 163.6, 156.4, 155.4, 142.7, 142.4, 141.3, 138.7, 129.8, 128.7, 125.5, 123.3, 122.3, 114.7, 114.6, 113.8, 111.3, 99.8, 97.7, 49.6, 45.5, 28.4, 27.6, 24.0, 20.7, 20.6, 10.5. HR-ESI-MS: m/z calcd. for HCA ($C_{28}H_{31}N_2O^+$, M^+), 411.2431; found, 411.2430.

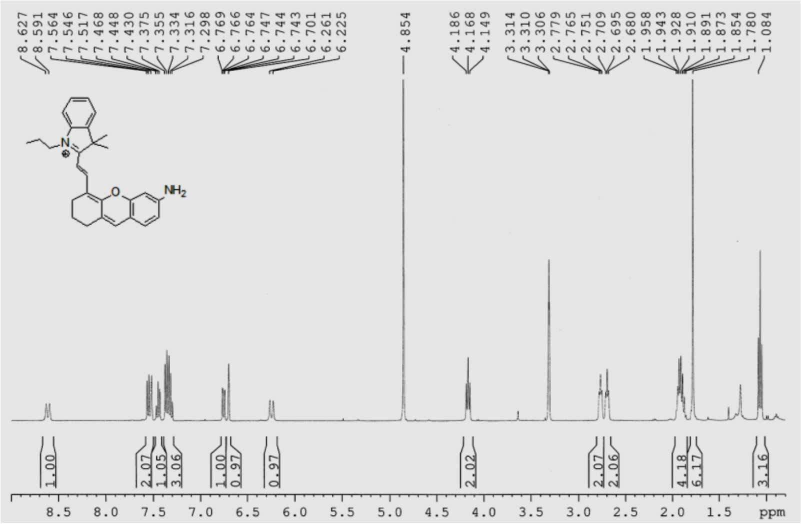

Figure S1. ^1H NMR spectrum of HCA (400 MHz, CD_3OD, 298 K).

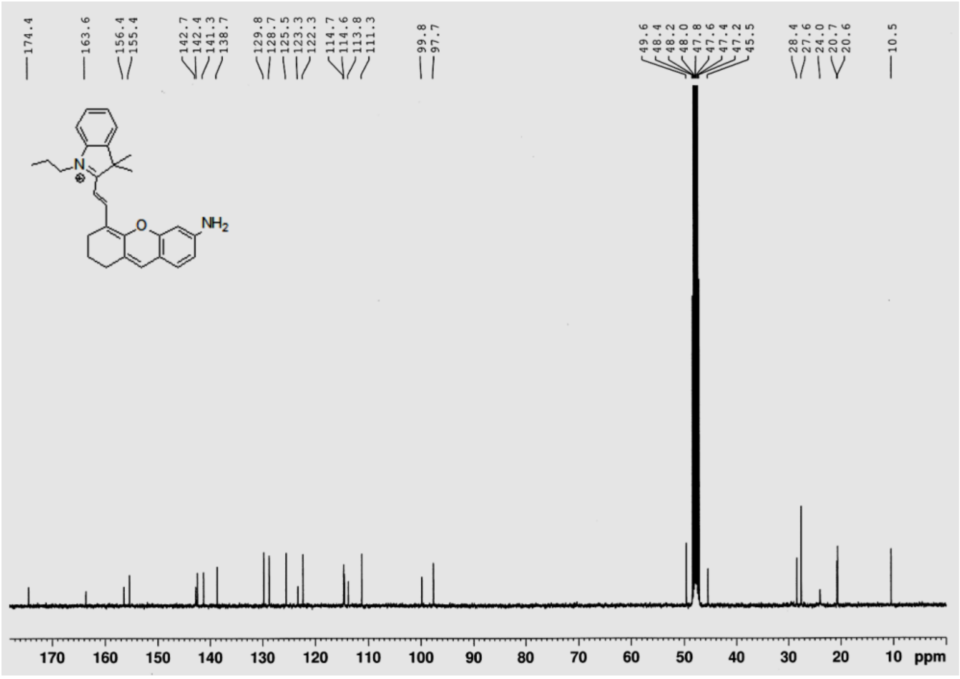

Figure S2. ^{13}C NMR spectrum of HCA (100 MHz, CD_3OD, 298K).

Synthesis of compound Boc-HCAL. Boc-L-leucine (440 mg, 1.6 mmol), HATU (760 mg, 2 mmol) and DIPEA (260 μL, 1.2 mmol) were dissolved in dry CH_2Cl_2 (40 mL) with stirring at 0 ℃ for 30 min. Then, HCA (164 mg, 0.4 mmol) in CH_2Cl_2 (5 mL) was introduced, and the reaction mixture was further stirred at room temperature for 4 h. The precipitate was filtered, and the filtrate was evaporated under reduced pressure. Then, the residue was purified by silica gel chromatography with CH_2Cl_2/CH_3OH (v/v, 100:1) as eluent, yielding compound Boc-HCAL as dark-blue solid (105 mg, yield 42%). ^1H and ^{13}C NMR spectra are shown in Figures S3 and S4. ^1H NMR (400 MHz, 298 K, $CDCl_3$): δ 8.90 (s, 1H), 8.65 (d, 1H, J = 14.4 Hz), 7.78 (s, 1H), 7.66 (s, 1H), 7.49 (d, 1H, J = 7.2 Hz), 7.43 (t, 1H, J = 7.6 Hz), 7.37 (t, 1H, J = 7.6 Hz), 7.28 (s, 1H), 7.24 (d, 1H, J = 8.4 Hz), 7.08 (s, 1H), 6.32 (d, 1H, J = 14.4 Hz), 5.39 (s, 1H), 4.34-4.29 (m, 1H), 4.23-4.19 (m, 2H), 2.67 (s, 2H), 2.61 (t, 2H, J = 5.6 Hz), 1.96-1.91 (m, 4H), 1.89 (s, 6H), 1.79 (m, 3H), 1.46 (s, 9H), 1.07 (t, 3H, J = 7.2 Hz), 0.98 (d, 6H, J = 6.4 Hz). ^{13}C NMR (100 MHz, 298 K, $CDCl_3$): δ 178.3, 172.9, 161.8, 153.6, 146.4, 142.6, 142.5, 141.5, 133.7, 129.0, 128.2, 127.4, 123.0, 117.8, 117.7, 114.7, 112.3, 106.5, 103.5, 80.3, 77.4, 55.0, 51.1, 46.8, 40.7, 29.3, 28.4, 28.2, 25.0, 24.0, 23.3, 21.5, 21.3, 20.4, 11.6. HR-ESI-MS: m/z calcd. for compound Boc-HCAL ($C_{39}H_{50}N_3O_4^+$, M^+), 624.3796; found, 624.3792.

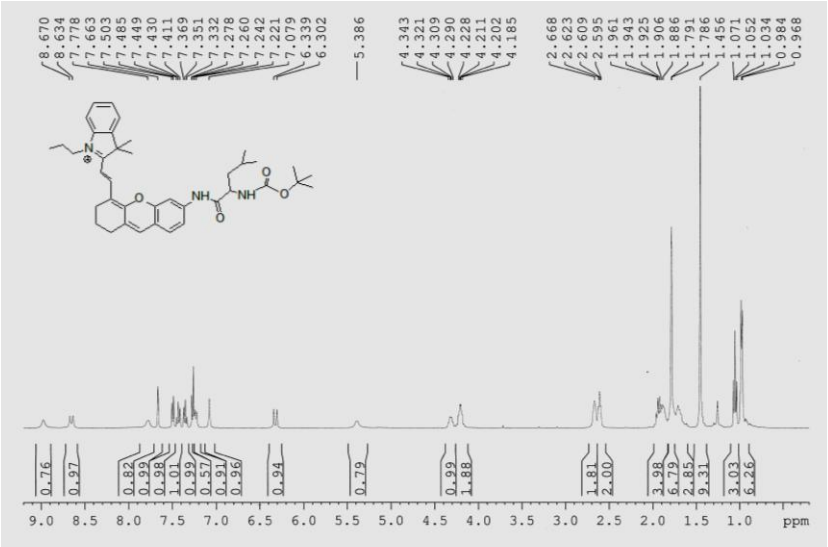

Figure S3. ^{1}H NMR spectrum of Boc-HCAL (400 MHz, $CDCl_3$, 298 K).

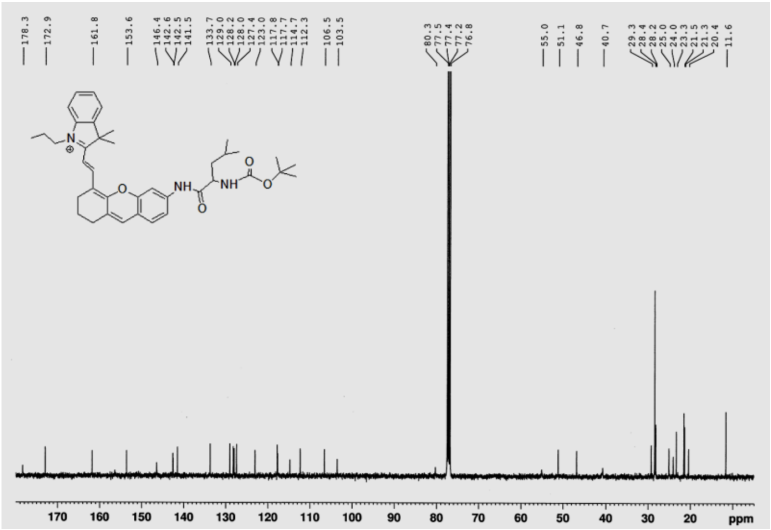

Figure S4. ^{13}C NMR spectrum of Boc-HCAL (100 MHz, $CDCl_3$, 298K).

Synthesis of probe HCAL. Compound Boc-HCAL (62 mg, 0.1 mmol) was stirred in CH_2Cl_2 (5 mL) containing trifluoroacetic acid (1 mL) at room temperature for 3 h. The solvent was removed by evaporation under reduced pressure, and the crude product was purified by silica gel chromatography eluted with CH_2Cl_2/CH_3OH (v/v, 10:1), affording probe HCAL as blue solid (44 mg, yield 84%). ^{1}H and ^{13}C NMR spectra are shown in Figures S5 and S6. ^{1}H NMR (400 MHz, 298 K, CD_3OD): δ 8.79 (d, 1H, J = 14.4 Hz), 7.98 (s, 1H), 7.67 (d, 1H, J = 6.8 Hz), 7.61 (d, 1H, J = 7.6 Hz), 7.57-7.54 (m, 1H), 7.53 (s, 2H), 7.49 (d, 1H, J = 7.6 Hz), 7.34 (s, 1H), 6.60 (d, 1H, J = 14.4 Hz), 4.38 (t, 2H, J = 7.2 Hz), 4.09 (t, 1H, J = 6.8 Hz), 2.80 (t, 2H, J = 5.6 Hz), 2.73 (t, 2H, J = 5.6 Hz), 2.00-1.92 (m, 4H), 1.84 (s, 9H), 1.09 (t, 3H, J = 7.2 Hz), 1.06 (d, 6H, J = 6.4 Hz). ^{13}C NMR (100 MHz,

298 K, CD_3OD): δ 179.9, 169.8, 162.4, 154.8, 147.5, 143.6, 143.0, 142.6, 133.6, 130.5, 130.3, 129.5, 128.8, 123.8, 120.0, 118.1, 115.9, 114.3, 107.4, 105.8, 53.9, 52.2, 47.8, 41.6, 30.2, 28.2, 25.5, 25.0, 23.3, 22.4, 21.9, 21.6, 11.6. HR-ESI-MS: m/z calcd. for probe HCAL ($C_{34}H_{42}N_3O_2^+$, M^+), 524.3272; found, 524.3272.

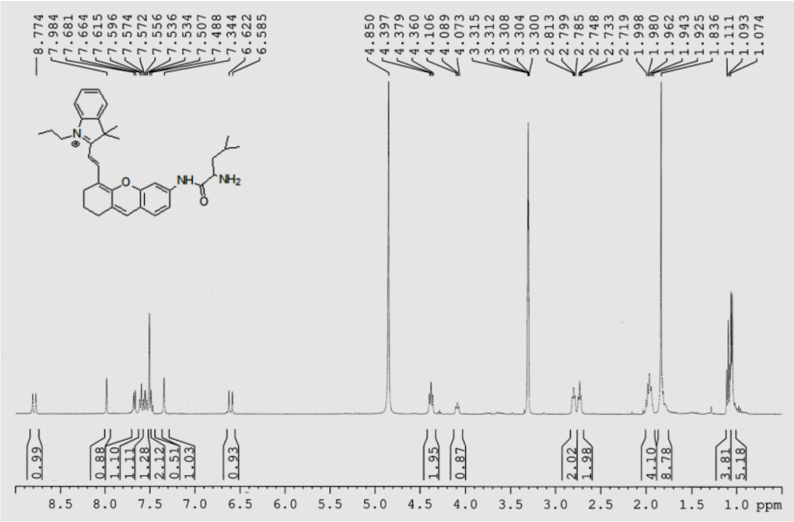

Figure S5. ^{1}H NMR spectrum of HCAL (400 MHz, CD_3OD, 298 K).

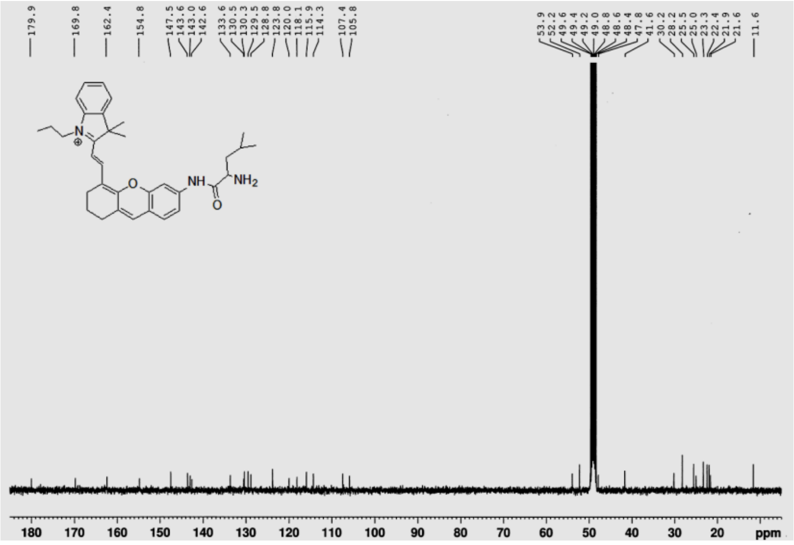

Figure S6. ^{13}C NMR spectrum of HCAL (100 MHz, CD_3OD, 298K).

3. General procedure for spectroscopic detection

Fluorescence quantum yield (Φ) was measured using ICG (Φ = 0.13 in DMSO, Licha et al, *Photochem. Photobiol.* **2000**, *72*, 392) as a standard according to the following equation:

$$\Phi_x = (\Phi_s \times A_s \times I_x \times \eta_x^2)/(A_x \times I_s \times \eta_s^2)$$

where A is the absorbance at the excitation wavelength (A is kept between 0.01 and 0.05),

I is the integrated area of emission spectra, and η is the refractive index of the solvent. The subscripts s and x refer to the standard and unknown, respectively.

The stock solution (1.0 mM) of probe HCAL was prepared in deoxygenated DMSO. In a test tube, 4 mL of PBS (pH 7.4) and 25 μL of 1 mM probe were mixed, followed by addition of LAP solution. The final volume was adjusted to 5 mL with PBS. After incubation at 37 ℃ for 90 min in a thermostat, the reaction solution was transferred to a quartz cell of 1-cm optical length to measure absorbance or fluorescence with $\lambda_{ex/em}$ = 670/705 nm. At the same time, solution containing no LAP was prepared and measured under the same conditions for comparison.

4. Cell incubation and fluorescence imaging

Cells (LO2 and HepG2) were grown on glass-bottom culture dishes (MatTek Co.) in DMEM supplemented with 10% (v/v) FBS and 1% (v/v) penicillin-streptomycin at 37 ℃ in a humidified 5% CO_2 incubator. Before use, the adherent cells were washed three times with FBS free DMEM. For LAP imaging, the cells were incubated with HCAL (5 μM) in DMEM at 37 ℃ for 30 min. Then cell imaging was conducted on an FV 1200-IX83 confocal laser scanning microscope with 635 nm excitation and 650-750 nm emission through a 100 × 1.4 NA objective. The pixel intensity of the cells in the fluorescence image was determined by using ImageJ software (version 1.45s, NIH). The cells can be taken as a Region of Interest (ROI), which is selected based on the periphery of the cells. For comparison, the pixel intensity at least from five cells in each fluorescence image was measured and averaged in this work.

For Ace stimulation, an appropriate concentration of Ace solution was added to the dishes containing adherent cells in DMEM supplemented with 10% (v/v) FBS and 1% (v/v) penicillin-streptomycin at 37 ℃ in a humidified 5% CO_2 incubator, and the cells were incubated for different periods of time.

5. Cytotoxicity assay

The cytotoxicity of HCAL to HepG2 cells was evaluated using standard MTT assay, as described previously (Song et al, *J. Mater. Chem.* **2012**, *22*, 12568).

6. In vivo imaging of mice

Ace was dissolved in warm saline (15 mg/mL). Five-week-old male BALB/c nude mice were obtained from Beijing Vital River Laboratory Animal Technology Co., Ltd. All animal operations were in accord with the institutional ethics committee regulations and

guidelines on animal welfare. According to the drug-induced liver injury model (Jaeschke et al, *Life Sci*. **2006**, *78*, 1670-1676), mice were fasted overnight and received an intraperitoneal injection of 300 mg/kg Ace between 8 and 9 am. After 6 h, the mice were subjected to an intravenous injection of 100 μL PBS containing 200 μM HCAL for in vivo imaging.

To establish HepG2 tumor model (Cui et al, *Food Funct.* **2016**, *7*, 455-463; Yang et al, *Cancer Lett.* **1997**, *17*, 93–98), 1×10^6 HepG2 cells suspended in PBS were injected on the shoulder of each mouse. After three weeks, tumor-bearing mice were injected by 50 μL PBS containing 50 μM HCAL for in vivo imaging. At different time intervals, the mice were anesthetized and scanned using an in vivo imaging system with a 650 nm excitation filter and a 700 nm emission filter.

7. Western blot analysis

HepG2 cells were lysed with RIPA buffer, and the cell lysates were diluted with PBS to obtain a solution of about 0.5 mg/mL of total proteins. The protein standard solution of 0.5 mg/mL was prepared. The protein concentration and western blot analyses were made following the previous method (Gong et al. *Chem. Sci.* **2016**, *7*, 788-792).

8. Supplementary figures

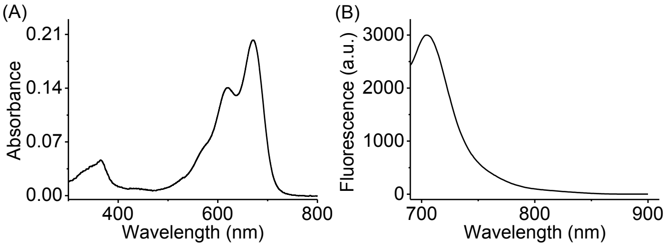

Figure S7. (A) Absorption and (B) fluorescence emission spectra of HCA (5 μM) in pH 7.4 PBS. λ_{ex} = 670 nm.

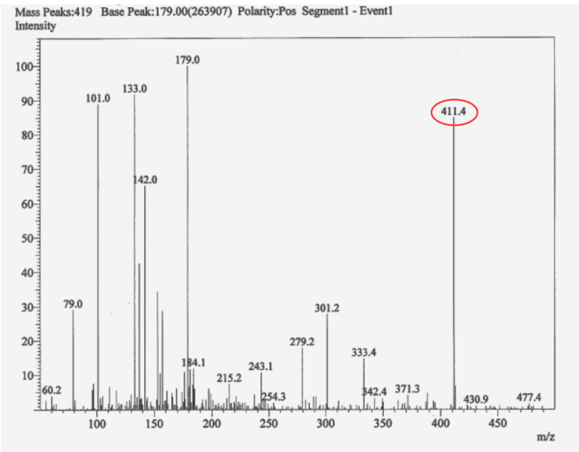

Figure S8. ESI-MS of the reaction solution of HCAL (50 μM) with LAP (500 U/L).

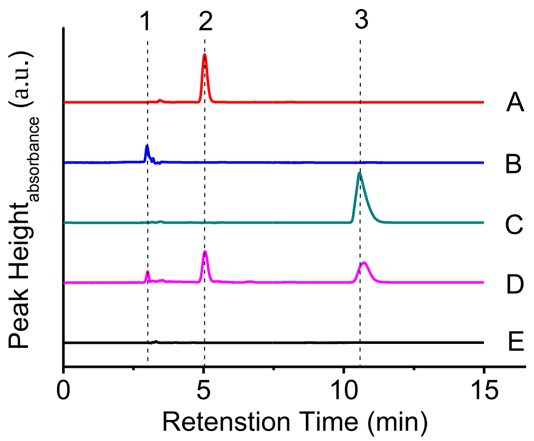

Figure S9. Chromatograms of different reaction systems. (A) HCAL (50 μM); (B) LAP (500 U/L); (C) HCA (50 μM); (D) the reaction solution of 50 μM HCAL with 500 U/L LAP for 30 min; (E) PBS (blank). The assignments of the peaks: (1) 2.98 min, LAP; (2) 5.03 min, HCAL; (3) 10.56 min, HCA.

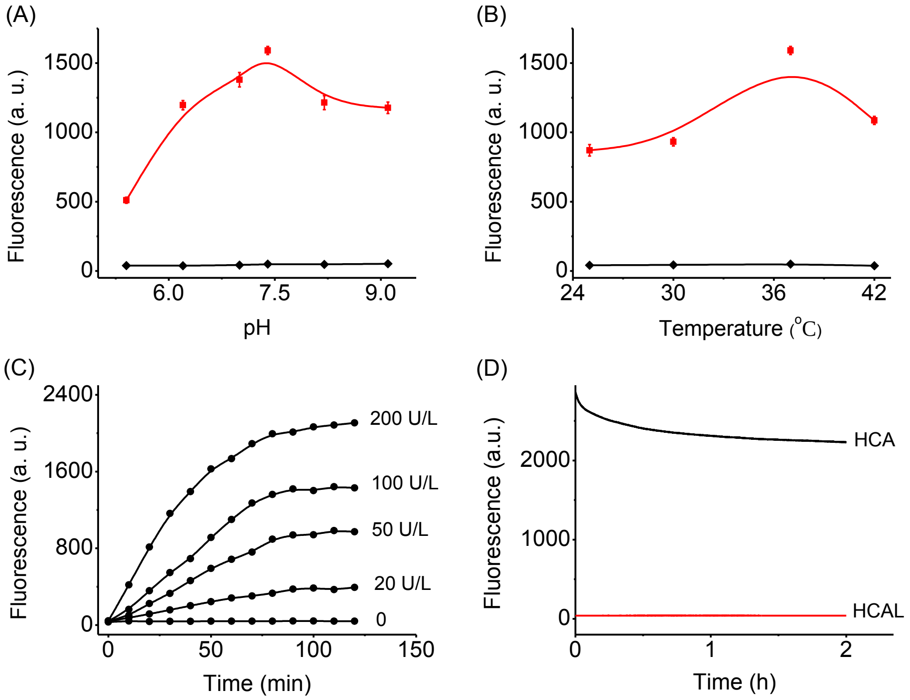

Figure S10. Effects of pH (A) and temperature (B) on the fluorescence of HCAL (5 μM) with (red) and without (black) LAP (100 U/L). (C) Plots of fluorescence intensity vs. the reaction time of HCAL (5 μM) with varied concentrations of LAP (0-200 U/L). $\lambda_{em/em}$ = 670/705 nm. (D) Photostability of HCAL and HCA under the irradiation of xenon lamp (150 W).

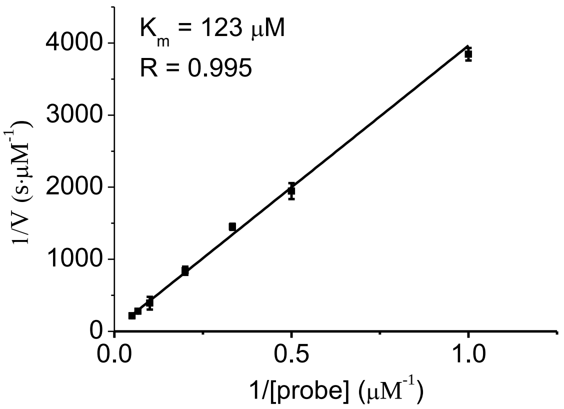

Figure S11. Lineweaver-Burk plot for the enzyme-catalyzed reaction. The Michaelis-Menten equation was described as: $V = V_{max}$ [probe]/(K_m + [probe]), where V is the reaction rate, [probe] is the probe concentration (substrate), and K_m is the Michaelis constant. Experiments were repeated three times at each probe concentration. Conditions: 30 U/L LAP, and 1-20 μM of HCAL. $\lambda_{ex/em}$ = 670/705 nm.

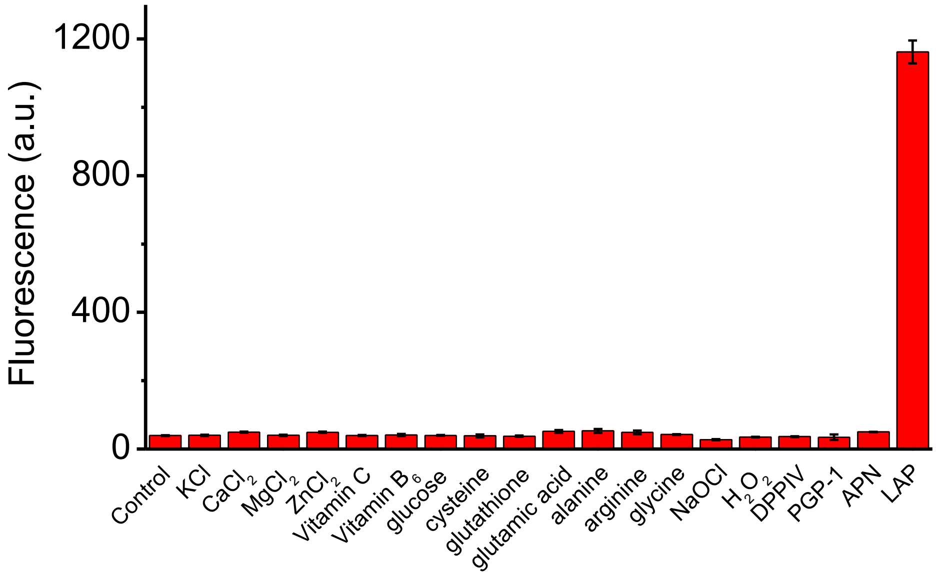

Figure S12. Fluorescence responses of probe HCAL (5 μM) to various potential interfering substances: (1) control; (2) KCl (150 mM); (3) $CaCl_2$ (2.5 mM); (4) $MgCl_2$ (2.5 mM); (5) $ZnCl_2$ (100 μM); (6) vitamin C (1 mM); (7) vitamin B_6 (1 mM); (8) glucose (10 mM); (9) cysteine (1 mM); (10) glutathione (1 mM); (11) glutamic acid (100 μM); (12) alanine (100 μM); (13) arginine (100 μM); (14) glycine (100 μM); (15) NaOCl (100 μM); (16) H_2O_2 (100 μM); (17) DPPIV (100 U/L); (18) PGP-1 (100 U/L); (19) APN (100 U/L); (20) LAP (50 U/L). $\lambda_{ex/em}$ = 670/705 nm.

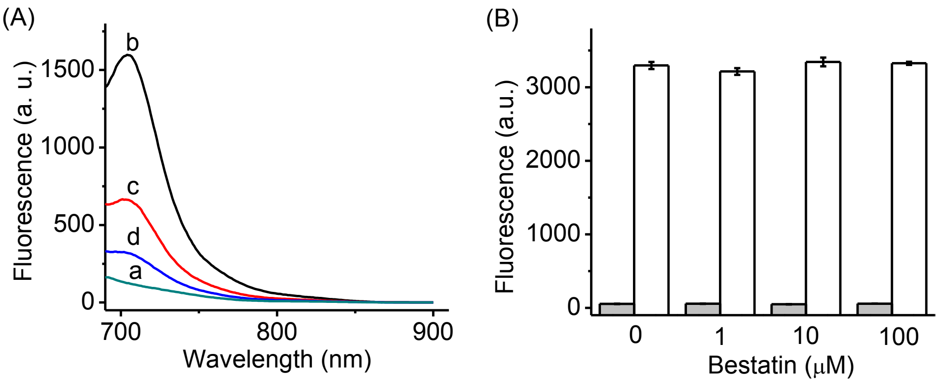

Figure S13. (A) Fluorescence emission spectra of different reaction systems. (a): HCAL (5 μM); (b): system (a) + LAP (100 U/L); (c): system (b) + bestatin (1 μM); (d): system (b) + bestatin (10 μM). (B) Effects of bestatin at varied concentrations on the fluorescence intensity of 5 μM HCAL (gray) or 5 μM HCA (white). $\lambda_{ex/em}$= 670/705 nm.

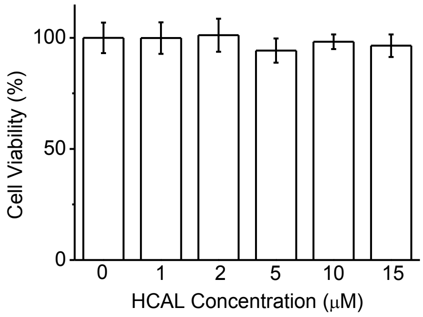

Figure S14. Effects of HCAL at varied concentrations (0-15 μM) on the viability of HepG2 cells. The viability of cells without HCAL is defined as 100%. The results are the mean ± standard deviation of five separate measurements.

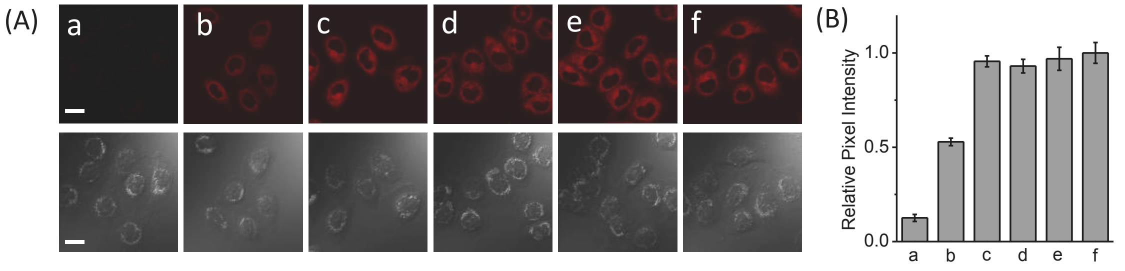

Figure S15. (A) Fluorescence images of LO2 cells incubated with HCAL (5 μM) for different periods of time (images a-f, 0, 15, 30, 45, 60 and 90 min). The differential interference contrast (DIC) images of the corresponding samples are shown below. Scale bars, 20 μm. (B) Relative pixel intensity of the corresponding fluorescence images in panel A (the pixel intensity from image f is defined as 1.0). The results are the mean ± standard deviation of three separate measurements.

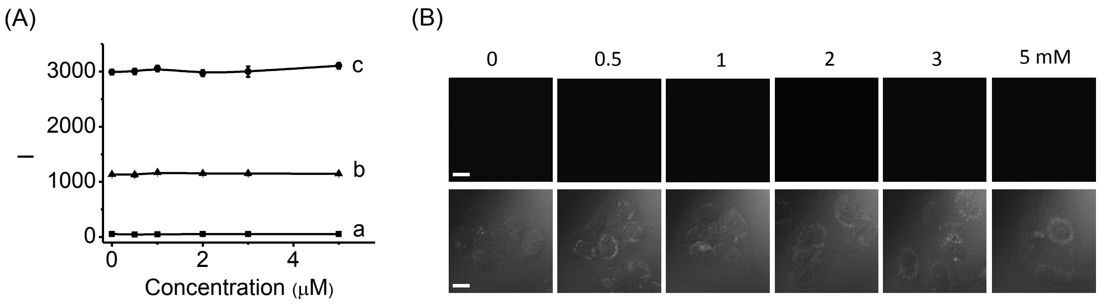

Figure S16. (A) The effects of Ace concentrations on the fluorescence intensity of different systems. (a): 5 μM HCAL; (b): 5 μM HCAL + 50 U/L LAP; (c): 5 μM HCA. (B) Confocal fluorescence images of LO2 cells treated with varied concentrations of Ace (0, 0.5, 1, 2, 3, and 5 mM) at 37 ℃ for 48 h. The DIC images of the corresponding samples are shown below. Scale bars, 20 μm.

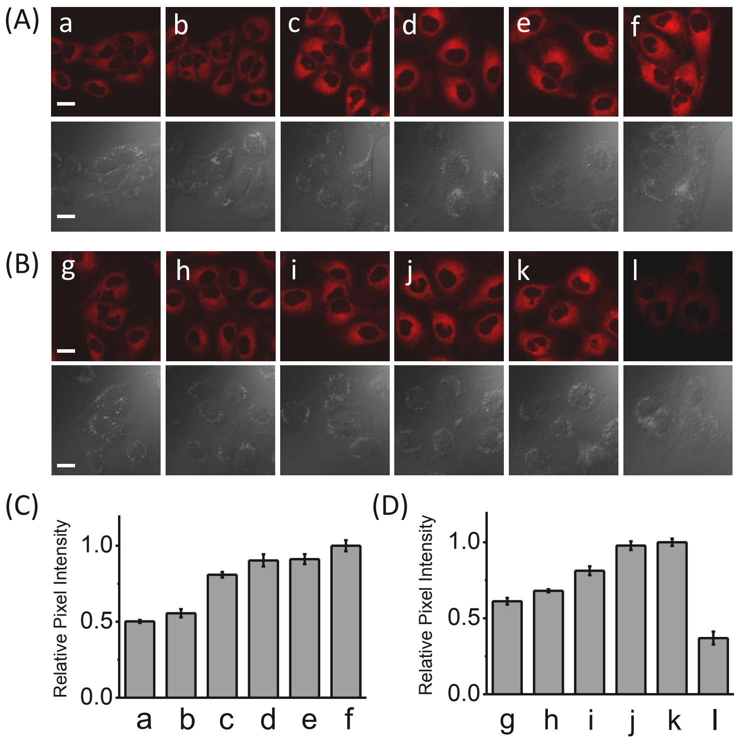

Figure S17. Effects of Ace on the LAP activity in LO2 cells. (A) Confocal fluorescence images of LO2 cells that were pretreated with varied concentrations of Ace (images a-f, 0, 0.5, 1, 2, 3, and 5 mM) for 48 h and then incubated with probe HCAL (5 μM). (B) Fluorescence images of LO2 cells that were pretreated with Ace (2 mM) for different periods of time (images g-k, 0, 12, 24, 48, and 72 h) and then incubated with probe HCAL (5 μM). Image l: LO2 cells that were first pretreated with Ace (2 mM) for 48 h, then with bestatin (100 μM) for 1 h, and finally incubated with probe HCAL (5 μM). The DIC images of the corresponding samples are shown below. (C) and (D) Relative pixel

intensity of the corresponding fluorescence images in panels A and B, respectively (the pixel intensity of the brightest image in each panel is defined as 1.0). Scale bars, 20 μm.

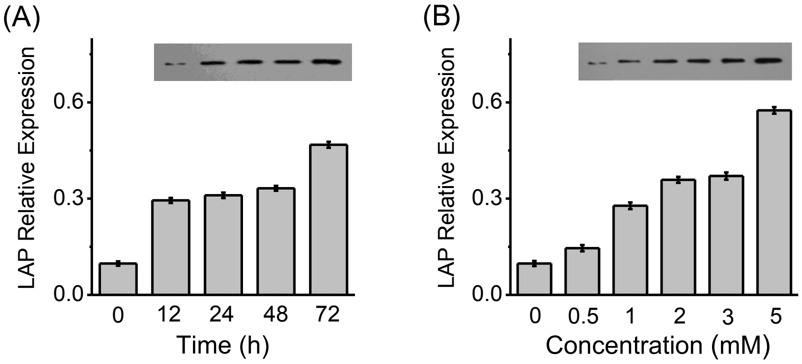

Figure S18. LAP levels in LO2 cells by western blot analyses. (A) LO2 cells were treated with Ace (2 mM) for different periods of time (0, 12, 24, 48, and 72 h). (B) LO2 cells were pretreated with varied concentrations of Ace (0, 0.5, 1, 2, 3, and 5 mM) for 48 h.

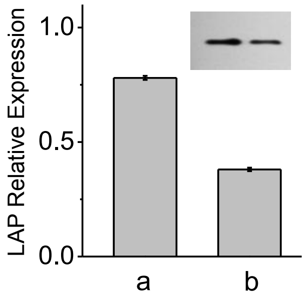

Figure S19. The LAP levels in HepG2 (a) and LO2 (b) cells by western blot analyses.

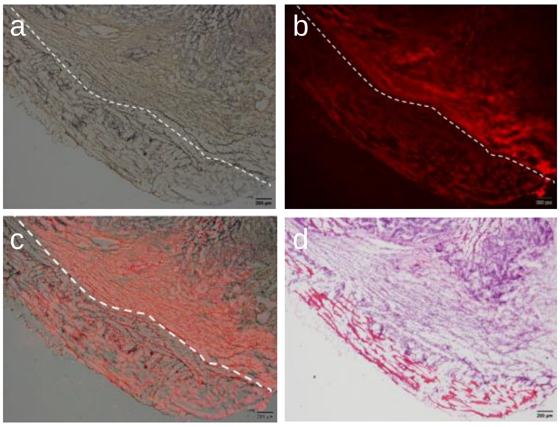

Figure S20. (a) Immunohistochemical staining of the specimen. (b) The fluorescence image of the specimen. (c) Overlay of immunohistochemical staining (a) and the fluorescence image (b). (d) Histological analysis of the resected specimen with hematoxylin-eosin staining. Scale bars, 200 μm.

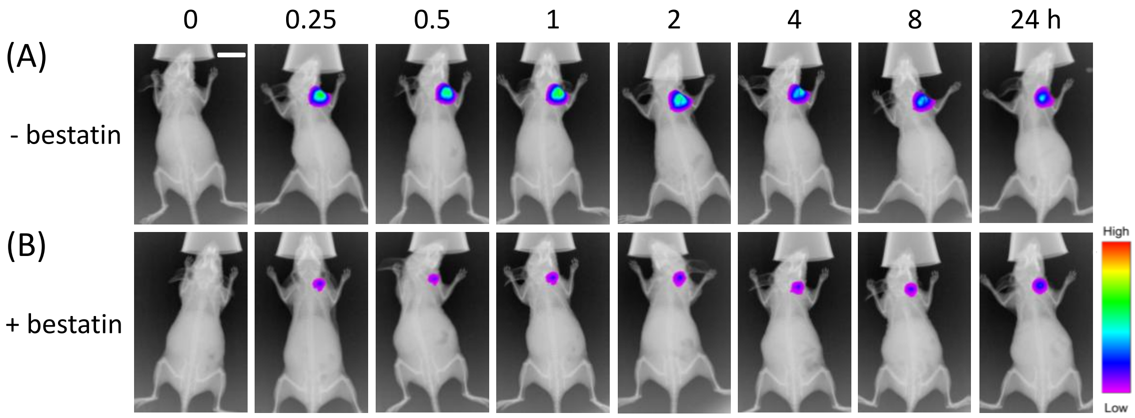

Figure S21. In vivo fluorescence imaging of HepG2 tumor xenografted mice. The mice were intratumorally preinjected with 50 μL of (A) PBS (control) and (B) bestatin (100 μM in PBS) for 1 h, and then subjected to intratumoral injection of 50 μL HCAL (50 μM) in PBS for different periods of time (0, 0.25, 0.5, 1, 2, 4, 8 and 24 h).
